# Randomness of Dengue Outbreaks on the Equator 

**DOI:** 10.3201/eid2109.141030

**Published:** 2015-09

**Authors:** Yirong Chen, Alex R. Cook, Alisa X.L. Lim

**Affiliations:** National University of Singapore, Singapore (Y. Chen, A.R. Cook, A.X.L. Lim);; National University Health System, Singapore (Y. Chen, A.R. Cook);; Yale–NUS College, Singapore (A.R. Cook)

**Keywords:** dengue epidemics, randomness, aseasonality, Singapore, vectorborne infections, viral infections, equator

## Abstract

A simple mathematical model without seasonality indicated that the apparently chaotic dengue epidemics in Singapore have characteristics similar to epidemics resulting from chance. Randomness as a sufficient condition for patterns of dengue epidemics in equatorial regions calls into question existing explanations for dengue outbreaks there.

Dengue, a vectorborne infectious disease, has complex epidemiologic dynamics (*1*). The recent expansion of the range of dengue makes this disease a considerable public health concern worldwide (*2*). In the city-state of Singapore, the number of dengue cases has increased dramatically since the 1990s, and all 4 serotypes of the dengue virus are endemic (*3*). Cyclical outbreaks of dengue of increasing magnitude have been observed with a cycle of 5–6 years (*4*), but this pattern appeared to cease in 2005, and no obvious cycle has occurred since then. Although other tropical and subtropical countries in Southeast Asia have distinct seasonality (*5*) so that dengue epidemics occur at distinct and predictable times of the year (*6*), Singapore’s proximity to the equator gives it an aseasonal climate, and the timing of dengue epidemics is irregular (*7**,**8*).

Many factors have been postulated to contribute to dengue’s spread in Singapore, such as a consistently warm and humid climate that favors year-round vector proliferation, high urbanization, and a tendency for vectors to live in human residences (*9*). The extent to which these factors affect dengue epidemics in aseasonal Singapore, if they do at all, is unclear. Competing explanations for the timing of large dengue outbreaks in Singapore can be found in the literature. One study attributes dengue epidemics to conducive temperatures and precipitation variations (*10*); another attributes them to variable maximum and minimum temperatures (*11*). Rainfall and temperature have been shown to be related to dengue outbreaks in Brazil, another equatorial country (*12*).

The tendency to see patterns where none exists has been well recognized. When 2 events happen contemporarily and a plausible story connects the events, the tendency to assume that 1 causes the other is strong (*13*). Cancer cases cluster around mobile phone masts (base stations), not because the radiation from a mast is carcinogenic at typical exposures but because numerous masts exist and occasionally cancer cases cluster together, similarly to spilled grains of rice (*14*). A study in the heuristics and biases program discusses a famous example from sports (*15*), which are notorious for stories being concocted around essentially chance outcomes. Basketball fans, coaches, and pundits often believe that players have “hot hand” streaks when they have a run of good form, making many shots in succession and playing above their usual level during a match. The study systematically deconstructed this belief by a series of statistical tests that showed that the patterns of actual hits and misses was consistent with mere chance—analogous to sequences of coin tosses rather than an illusory hot hand (*15*).

In probabilistic models, chance is represented by error terms, or noise, encompassing all the many complicating factors that are not worth including in the systematic signal. Past models for dengue in Singapore have accounted for chance alongside systematic effects of the weather and other factors (*10**,**11*). However, is chance alone sufficient to explain the frequent, large, and ostensibly chaotic outbreaks we observe? We sought to assess whether the rise and fall of dengue outbreaks from week to week in Singapore come in runs or are indistinguishable from random noise and thereby whether it is necessary to consider other possible drivers of these epidemics.

## The Study

We reviewed data on the weekly incidence of clinically diagnosed dengue in Singapore during 2003–2012. We compared the number of dengue cases per week to a simple simulation model (Technical Appendix) with no environmental drivers other than the dependence of weekly number of cases from up to 4 weeks before. Summaries of observed incidence and of the simulated aseasonal model were compared for assessing proximity of the behavior of observed cases to the behavior of simulated cases.

**Figure 1:**
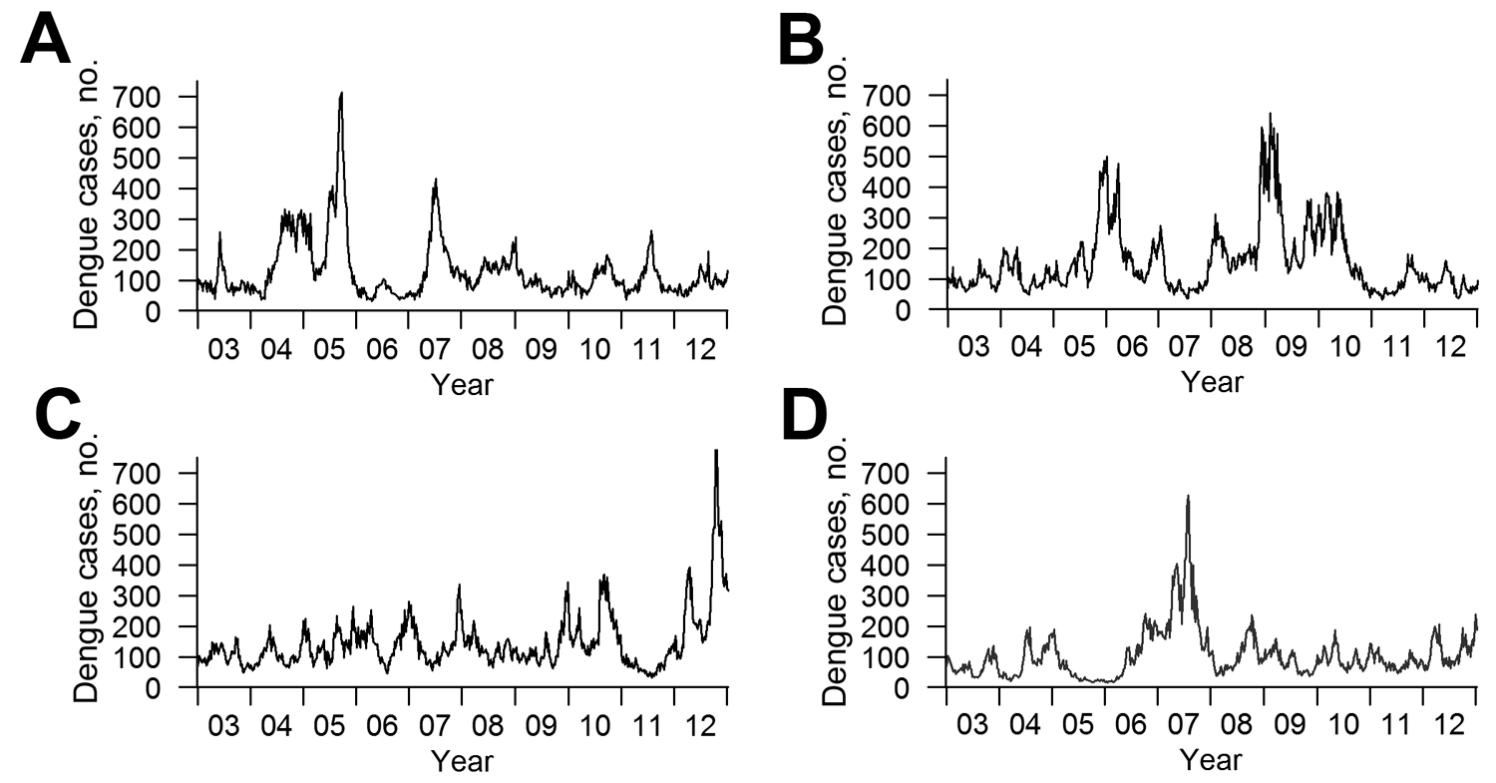
Weekly trends for observed and simulated dengue incidence, 2003–2012, Singapore. A) Weekly trends for the actual scenario of observed dengue incidence. B–D) Three randomly generated simulated scenarios from the aseasonal model described in the text and the Technical Appendix. Although the peaks are not synchronized, similar patterns can be discerned; large and small outbreaks of similar scale and frequency occur in all 4 scenarios.

The simulation model used was a standard autoregressive time series model in which the number of cases during any week affects the mean number of cases for the 4 weeks that follow. We allowed the simulated number to have a random variation around that mean; data were log-transformed to ensure that incidence was positive. The fitted autoregressive model was used to simulate synthetic dengue outbreaks over multiple decades, and incidence of simulated outbreaks was compared with observed incidence. We devised a series of statistical measures that were inspired by the “hot hand” in basketball study (*15*) and that might falsify the model that accounted for chance alone. This model included correlation between dengue incidence by week and the preceding week (the autocorrelation function), the probability distribution for the weekly incidence aggregated over 10 years, the distribution of the annual number of cases, the maximum number of cases observed over the previous decade, and the probability of a rise in incidence each week following a series of rises (i.e., the possible beginning of an epidemic) or a series of declines (i.e., the possible ending of an epidemic). We also created simulated trajectories (Figure 1).

## Conclusions

**Figure 2:**
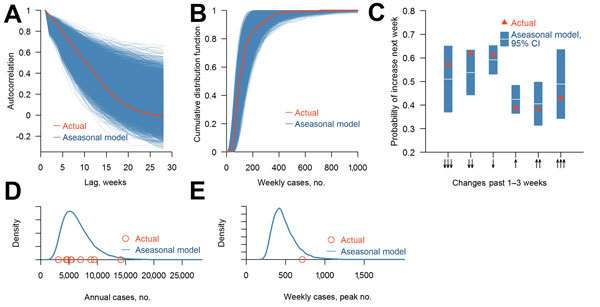
Comparison of observed dengue incidence and incidence from simulated aseasonal models, 2003–2012, Singapore. A) Distribution of actual and simulated autocorrelation functions at different time lags (e.g., this week versus next week; last week versus next week, etc.) B) Distribution of cumulative distribution function of the simulated weekly number of dengue cases and cumulative density function of the actual numbers of cases. C) Conditional probabilities of an increase in number of dengue cases and 95% CIs for simulated scenario and actual data, given 1–3 consecutive decreases or increases. D) Density plot of simulated and actual annual number of dengue cases. E) Density plot of simulated 10-year maximum number of cases and actual 10-year number of cases.

For all metrics considered, the actual scenario (i.e., the observed dengue incidence) was fully consistent with the aseasonal model; both the autocorrelation function (Figure 2, panel A) and the cumulative probability of dengue incidence (Figure 2, panel B) from the historical incidence data lie within the distribution resulting from the aseasonal model. The probabilities of an increase in incidence each week that follows a series of rises or falls and corresponding 95% CIs calculated on the basis of simulations from the aseasonal model all include the proportions observed historically (Figure 2, panel C). Furthermore, the distribution of the annual incidence (Figure 2, panel D) and the maximum observed incidence over the decade (Figure 2, panel E) are consistent with the aseasonal model. Similarly, the number of successive increases or decreases over the decade was consistent with chance (p = 0.18).

These metrics are not conventional measures of dengue surveillance data; they capture more complex, emergent properties of the epidemic process. However, our findings show that, for dengue incidence in equatorial Singapore, where average monthly temperatures vary only from 26°C–28°C, randomness alone is sufficient to explain the apparent epidemics of dengue. Although seasonal factors may have a role, as the literature suggests (*10**,**11*), seasonality or other temporal drivers such as fluctuation in the intensity of the country’s vector control program are not necessary to explain the qualitative and quantitative patterns of dengue in this equatorial city-state. As our results suggest, the possibility that dengue outbreaks occur in aseasonal locations because of chance should be considered. 

Technical Appendix Statistical modeling approach used for aseasonal modeling of dengue incidence in Singapore.
