# Supplementary material for: Randomness of Dengue Outbreaks on the Equator
Source: Emerg Infect Dis. 2015 Sep;21(9):1651–3. doi: 10.3201/eid2109.141030 (PMC4550167; doi:10.3201/eid2109.141030)
Supplement: Technical Appendix — Statistical modeling approach used for aseasonal modeling of dengue incidence in Singapore. [file 14-1030-Techapp-s1.pdf]

DOI: http://dx.doi.org/10.3201/eid2109.141030

Randomness and Dengue Outbreaks on the Equator

Technical Appendix

Statistical Modeling Approach

The aseasonal model described in the paper belongs to a group of regression models called *autoregressive models*. In these models, the number of dengue cases during 1 week is regressed upon the incidence in preceding weeks. The order of the model (i.e., the number of preceding weeks to autoregress upon) is selected to ensure that there are no further correlations between the model "errors," or differences between observed and predicted numbers of cases. This process of selecting the order of the model was done by using the Portmanteau, or Ljung-Box, statistical test (*1*), which suggested the number of dengue cases needed to depend on the weekly number of cases during the previous 4 weeks to meet this statistical requirement. (Using a simpler model that depended on just the previous week did not substantively change the findings in the paper.) To account for incidence necessarily being positive, we used the standard approach of logging the number of dengue cases before modeling them; this approach had a secondary benefit of making the variability more stable. Letting the number of cases in week t be D_t, we therefore calculated and worked with $y_t = \ln(D_t)$. The fitted model was

$$y_t = 0.29 + 0.62y_{t-1} + 0.32y_{t-2} + 0.13y_{t-3} - 0.14y_{t-4} + e_t$$

where e_t is random noise assumed to follow a normal distribution with mean 0 and SD 0.22 (the latter estimated from the data). These estimates were obtained by using the standard regression method of least-squares. The model therefore captures the decay in the risk of secondary cases over the 4 weeks after infection.

The fitted model was used to simulate synthetic datasets that, because the model contains no seasonal forcing, are governed purely by randomness (the e_t terms) and short-term contagion (the y_{t-1} to y_{t-4} terms) but by no other drivers. Three examples are provided in Figure 1 in the main text. From these simulated data, which covered thousands of hypothetical decades of

dengue in Singapore, summaries were extracted that could be compared to the analogous summary from reality. These summaries provide a way to falsify the aseasonality model if the actual summary falls outside the range from the simulations. The summaries were inspired by metrics used in "the hot hand in basketball" perception of randomness study (*2*) but adapted to the context of weekly case counts of dengue. These metrics included the following:

1. **The autocorrelation function.** This function is the correlation between the (log transformed) number of cases for 1 week against the number k weeks later (i.e., between y_t and y_{t+k}). This function drops from 1 for small lags k to 0 for large lags. The speed at which it drops indicates the degree of memory in the dengue time series (e.g., if there is a high incidence of dengue 1 week, there should be a high incidence during the following week). Each simulation yielded a single curve, over which the curve for the data was overlaid.

2. **The cumulative probabilities.** For a particular number of cases, say D, this metric is the proportion of weeks with D or fewer cases. It therefore captures the overall distribution of dengue incidence but more smoothly than a histogram would. Again, each simulation yielded a single curve, over which the curve for the data was overlaid.

3. **The conditional probability of a rise** in the number of dengue cases from 1 week to the next (t to $t + 1$), given that a rise occurred between week $t - 1$ and week t. Similar probabilities given 2 or 3 successive rises, or 1 to 3 falls, were also considered. These probabilities were calculated directly from the simulations, summarized with a 95% CI, and the corresponding proportions in the historical time series overlaid.

4. **The yearly number of cases.** The distribution from simulations was summarized by using the statistical technique called *kernel density estimation*, and the 10 yearly counts from the data were overlaid.

5. **The maximum weekly number of cases over 10 years.** Again, simulated maxima were summarized by using kernel density estimation, with the maximum weekly incidence observed over the decade in question overlaid as a point.

We also created a further statistical test that counted the number of *runs* (i.e., the number of weeks of successive rises or falls in dengue incidence). The distribution of the number of runs was obtained from the set of simulated datasets and used to generate a p value for the hypothesis that the aseasonal model generated the observed dataset.

The rationale for these summaries is that they capture more complex properties that emerge from the simple aseasonal model described above but that should not concord with the data if seasonal drivers were needed to explain the patterns observed. For instance, with clear seasonal epidemics, following a run of week-on-week rises in incidence, a further rise would be noticeably more likely than 50%, whereas a random process (like the aseasonal model) would see this probability much closer to 50%.

In subtropical Taiwan, for example, there is clear seasonality, which is reflected in cycles in dengue epidemics (Technical Appendix Figure 1, panel A). Simulations from the aseasonal model (Technical Appendix Figure 1, panels B–D) fail to capture both the seasonality and size of dengue outbreaks. The 5 measures we developed were able to indicate clear discrepancies between the data and the simulations (Technical Appendix Figure 2, panels A and C). For equatorial Singapore, however, for all the characteristics considered, the historic data were consistent with every metric we devised to test the aseasonal model:

1. For the autocorrelation function (Technical Appendix Figure 2, panel A), the actual autocorrelation falls near the middle of the distribution of simulations under the aseasonal model for all weeks considered, up to the point when the autocorrelation reached 0 (around 6 months). The close correspondence between simulated and observed autocorrelation functions indicates that the random, aseasonal model exhibits the same degree of *memory* as the actual time series; that is, following an epidemic peak, the outbreak reverts to endemic levels at the correct pace; from a trough, epidemics occur at the correct speed.
2. Similarly, the observed cumulative probability function (Technical Appendix Figure 2, panel B) falls near the middle of the distribution of model predictions for all values of the weekly incidence up to the maximum weekly incidence observed. This function indicates that the aseasonal model gives the correct frequency and size of endemic and epidemic phases.

3. The conditional probabilities of rises in the weekly number of cases from 1 week to the next given runs of rises or falls (Technical Appendix Figure 2, panel C) was also consistent between data and model and in the opposite direction to what would be expected given seasonal forcing, with a fall more likely to follow a rise and vice versa. With seasonal forcing, we would expect positive correlations in weather conditions from 1 week to the next to translate to positive correlations in epidemic growth or decline.
4. The 10 annual incidences (Technical Appendix Figure 2, panel D) were fully consistent with the distribution from the model predictions. These incidences indicate that the aseasonal model gives annual tallies that are consistent with those we observed.
5. The peak number of weekly cases over 10 years (Technical Appendix Figure 2, panel E) is similarly within the range of plausible scenarios under the aseasonal model.

The test for the number of runs did not find any deviation from the aseasonal model ($p = 0.18$), indicating that the consistency of epidemic rise and fall from 1 week to the next also agrees with the absence of seasonality. Further, simulated epidemics (Technical Appendix Figure 1) expressed qualitatively similar behavior to the historic patterns, with similar magnitude and frequency of epidemics and similar endemic behavior.

References

<jrn>1. Ljung G, Box G. On a measure of lack of fit in time series models. Biometrika. 1978;65:297–303.</jrn>

<jrn>2. Gilovich T, Vallone R, Tversky A. The hot hand in basketball: on the misperception of random sequences. Cognit Psychol. 1985;17:295–314.</jrn>

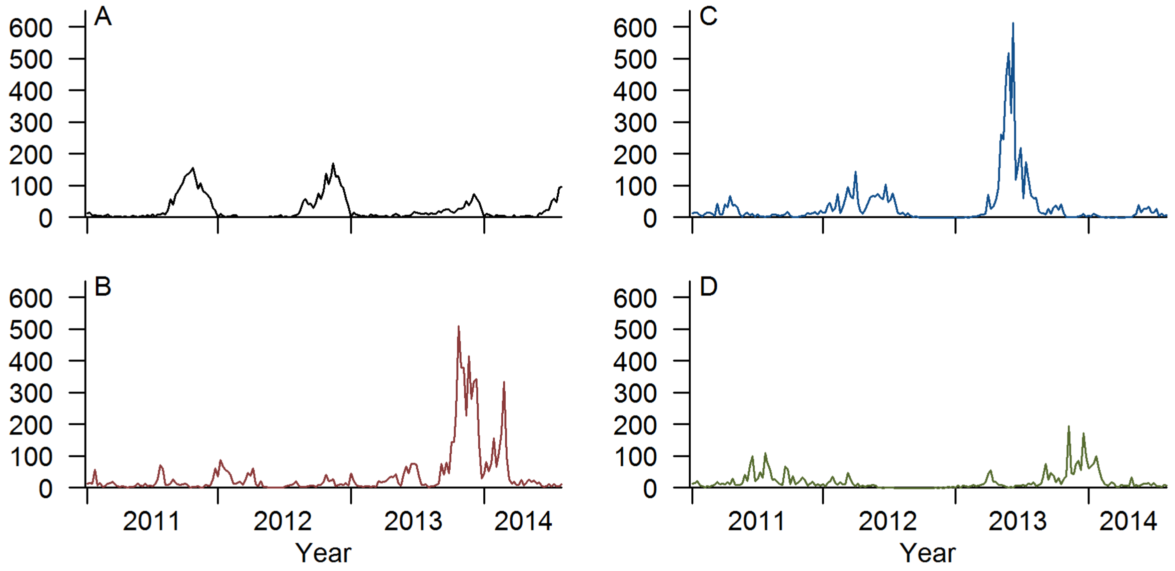

Technical Appendix Figure 1. The aseasonal model applied to Taiwan (2011–2014). A) Dengue trend over >3 years in Taiwan. Clear seasonality can be observed, and epidemics tend to appear at year end. B–D) Aseasonal simulation models. The timing and size of outbreaks differ markedly from the actual scenario of observed dengue incidence.

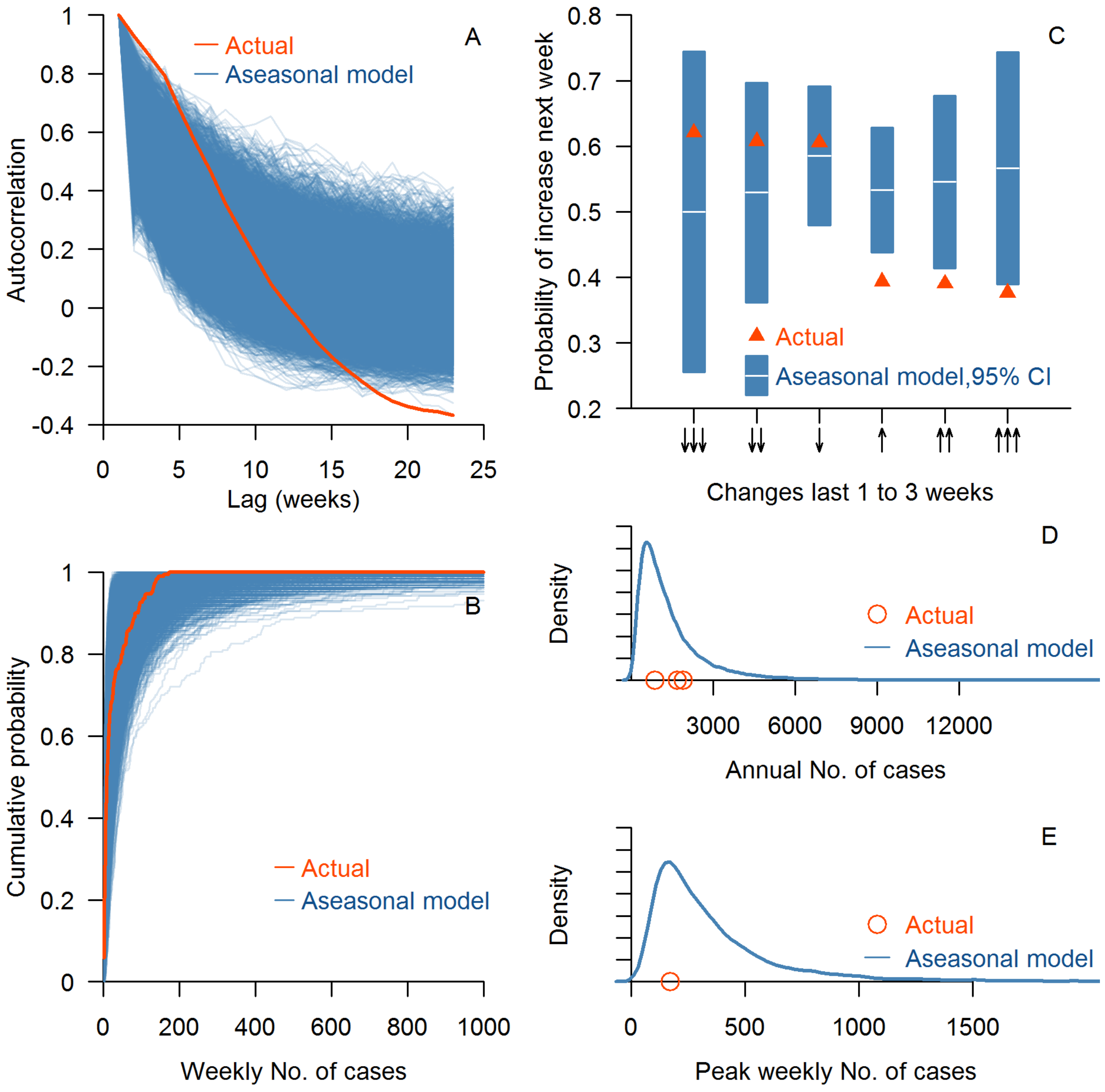

Technical Appendix Figure 2. Comparison of observed dengue incidence and the simulated aseasonal model in Taiwan. A) Distribution of simulated autocorrelation functions and actual autocorrelation functions at different lags. B) Distribution of cumulative density function of the simulated weekly number of cases and cumulative density function of the actual numbers. C) Conditional probabilities of an increase in number of dengue cases and 95% CIs for simulated scenario and actual data, given 1–3 consecutive decreases or increases. D) Density plot of simulated yearly number of cases and actual number. E) Density plot of simulated 10-year maximum and actual number.
